# Overactive bladder syndrome symptoms in youth with abdominal pain-associated disorders of gut–brain interaction

**DOI:** 10.1038/s41598-023-37937-w

**Published:** 2023-07-08

**Authors:** Hunter J. Friesen, Pierce Thompson, Jennifer V. Schurman, Jennifer M. Colombo, Craig A. Friesen

**Affiliations:** Division of Gastroenterology, Hepatology, and Nutrition, Children’s Mercy Kansas City, Kansas City, MO 64108 USA

**Keywords:** Gastroenterology, Medical research

## Abstract

The purpose of the current study was to assess the frequency of overactive bladder syndrome (OBS) symptoms and their relationship to gastrointestinal symptoms in youth with abdominal pain-associated disorders of gut–brain interaction (AP-DGBI). This is a retrospective study of 226 youth diagnosed with an AP-DGBI. As part of standard care, all patients completed a symptom questionnaire regarding gastrointestinal and non-gastrointestinal symptoms including increased urinary frequency, nighttime urination, and urinary urgency. Overall, 54% of patients reported at least one OBS symptom. Increased frequency of urination was reported by 19%, urinary urgency by 34%, and nighttime urination by 36%. Increased frequency of urination and urinary urgency were associated with a change in stool form, a change in stool frequency, and in those fulfilling criteria for IBS. Increased frequency of urination was reported more frequently in those reporting predominantly loose stools (33% vs. 12%). Urinary symptoms are common in youth with AP-DGBI. Increased urinary frequency and urinary urgency are specifically associated with IBS, with increased urinary frequency being primarily associated with diarrhea predominant IBS. Further studies are needed to determine the impact of OBS on AP-DGBI severity and quality of life, and whether they impact DGBI treatment.

## Introduction

Chronic abdominal pain is quite common in children and adolescents^1^. The majority fulfill criteria for an abdominal pain-associated disorder of gut–brain interaction (AP-DGBI), with the most common being irritable bowel syndrome (IBS) and functional dyspepsia (FD)^2–4^. IBS is defined by the presence of abdominal pain in association with a change in stool frequency, a change in stool form, or a change (increase or decrease) in pain with a stool^2^. FD is defined by the presence of epigastric pain or burning, early satiety, or postprandial bloating^2^.

It is common for patients to fulfill criteria for both IBS and FD (i.e., FD/IBS overlap), particularly with the evolution to Rome IV criteria^5–7^. Furthermore, IBS and FD have been shown to overlap with other disorders such as gastroesophageal reflux (GERD) and overactive bladder syndrome (OBS)^7,8^. OBS is defined by three primary symptoms: increased urinary frequency, nighttime urination, and urinary urgency^9^. OBS has been associated with both IBS and FD in adults^10–13^. OBS has been associated with constipation in children and has been previously shown to be common in youth fulfilling FD criteria^7,14–17^. To our knowledge, associations of OBS symptoms with other gastrointestinal symptoms or more broadly across specific AP-DGBI diagnoses have not been previously reported for children and adolescents.

Evaluating relationships between OBS and gastrointestinal symptoms may have implication for understanding shared pathophysiology and may further delineate patient symptom heterogeneity within AP-DGBI diagnostic categories. The purpose of the current study was to assess the frequency of increased urinary frequency, nighttime urination, and urinary urgency in youth with AP-DGBI and to determine whether they are associated with specific gastrointestinal symptoms or AP-DGBI diagnosis.

## Results

A total of 226 patients were evaluated. The mean age was 13.9 ± 2.7 years and 72% were female. The frequency of pain was daily in 62%, several times/week in 21%, and weekly in 17%. The frequency of individual gastrointestinal symptoms is shown in Table 1.

**Table 1:** Frequency of individual gastrointestinal symptoms in pediatric patients with abdominal pain-associated disorders of gut–brain interaction (N = 226).

Symptom	Frequency (%)
Pain with eating	64
Early satiety	64
Postprandial bloating	62
Nausea	91
Vomiting	31
Heartburn	43
Change in stool frequency	32
Change in stool form	40
Change in pain with stool	38
Excess gas	38

Rome IV criteria were fulfilled for FD by 89% and for IBS by 59% of patients; 52% fulfilled criteria for both FD and IBS. Reported stool frequency was 1–2 times daily in 62% of patients; 18% reported stooling less than daily and 21% reported having 3 or more stools daily. Predominantly hard stools were reported by 17% and predominantly loose stools were reported by 33%. No change in pain with stools was reported by 62% of patients while 24% reported decreased pain with stools and 14% reported increased pain with stools.

**Figure 1:**
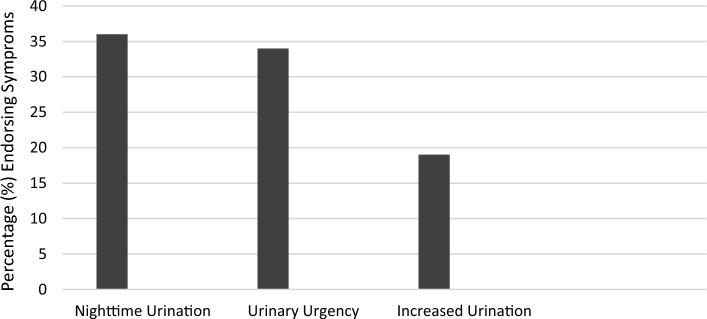
Frequency of overactive bladder symptoms in pediatric patients with abdominal-pain associated disorders of gut–brain interaction (N = 226).

Overall, 54% of patients reported at least 1 overactive bladder symptom. The frequency of individual overactive bladder symptoms is shown in Fig. 1.

There were no significant differences between females and males with regards to increased urination (19% vs. 19%), nighttime urination (39% vs. 30%), or urinary urgency (36% vs. 28%). There were no significant differences between adolescents (≥ 13 years of age) and children with regards to increased urination (20% vs. 18%), nighttime urination (34% vs. 40%), or urinary urgency (34% vs. 33%).

### Increased urination

Increased urination was reported more frequently in those fulfilling IBS criteria (25% vs. 10%; OR 3.14, 95%CI 1.42–6.91. Specifically, increased urination was reported more frequently in those reporting a change in stool form (32% vs. 10%; OR 4.04, 95%CI 1.99–8.20) or a change in stool frequency (38% vs. 10%; OR 5.18, 95%CI 2.56–10.46). The frequency of increased urination did not differ statistically between those fulfilling FD criteria as compared to those who did not (20% vs. 12%; OR 1.82, 95%CI 0.52–6.39). Increased urination was reported by 33% of those reporting predominantly loose stools vs. 12% of those not reporting predominantly loose stools (OR 3.82, 95%CI 1.90–7.69). Increased urination was reported by 15% of those reporting predominantly hard stools vs. 20% of those not reporting predominantly hard stools (OR 0.74, 95%CI 0.29–1.90).

### Nighttime urination

There was no significant difference in the frequency of reporting nighttime urination in those reporting a change in stool form (41% vs. 40%), a change in stool frequency (37% vs. 29%), or those fulfilling criteria for IBS (37% vs. 35%) or FD (36% vs. 32%). Nighttime urination was reported by 44% of those reporting predominantly loose stools vs. 32% of those not reporting predominantly loose stools (OR 1.67, 95%CI 0.94–2.97). Increased urination was reported by 38% of those reporting predominantly hard stools vs. 36% of those not reporting predominantly hard stools (OR 1.14, 95%CI 0.56–2.31).

### Urinary urgency

Urinary urgency was reported more frequently in those fulfilling IBS criteria (42% vs. 22%; OR 2.59, 95%CI 1.42–4.72) Specifically, urinary urgency was reported more frequently in those reporting a change in stool form (43% vs. 27%; OR 1.99, 95%CI 1.13–3.48) or a change in stool frequency (47% vs. 27%; OR 2.39, 95%CI 1.33–4.27). The frequency of urinary urgency did not differ statistically between those fulfilling FD criteria as compared to those who did not (35% vs. 24%; OR 1.69, 95%CI 0.65–4.43). Urinary urgency was reported by 41% of those reporting predominantly loose stools vs. 30% of those not reporting predominantly loose stools (OR 1.65, 95%CI 0.92–2.94). Increased urination was reported by 44% of those reporting predominantly hard stools vs. 32% of those not reporting predominantly hard stools (OR 1.67, 95%CI 0.82–3.37).

## Discussion

OBS symptoms, particularly nighttime urination and urinary urgency, are common in youth with AP-DGBIs. The bladder may represent a potential source of pain given the frequency of patients reporting suprapubic pain, particularly in those reporting increased frequency of urination in the current study. OBS symptoms are associated with IBS, specifically diarrhea predominant IBS (IBS-D). Both increased frequency of urination and urinary urgency, but not nighttime urination, were associated with a change in stool frequency and form and increased urinary frequency was associated with predominantly loose stools. Hard stools did not increase the risk of OBS symptoms; this was somewhat surprising given the known association between constipation and OBS symptoms in children^14–17^. OBS symptoms have been associated with IBS in most, but not all, adult studies^10–12,18^. While OBS symptoms are also seen frequently in children with FD in the current and previous studies, they did not occur more frequently in those with FD as opposed to those without^7^. Additionally, we found no relationship between OBS symptoms and any specific FD symptoms. Previous adult studies have reported a relationship between OBS symptoms and FD but with a weaker association than that for IBS^11–13^.

The association of IBS and diarrhea with OBS symptoms would suggest shared pathophysiologic mechanisms or possibly visceral organ cross-sensitization. Shared pathophysiologic mechanisms may include hypersensitivity, inflammation (including allergy), an altered microbiome, altered epithelial function, and psychologic dysfunction. Visceral hypersensitivity with upregulation of TRPV1 channels is considered a key process in IBS^19,20^. OBS is associated with increased neural sensitivity and upregulation of TRPV1 has been reported in several studies^9,21–23^. Histologic inflammation also has been implicated in both OBS and IBS^9,24,25^. Both are associated with allergies and mast cells have been specifically implicated in both conditions and may be associated with increased visceral hypersensitivity^9,20,24,26–32^. Mast cell-derived histamine may be important in this process as it upregulates TRPV1 and can enhance activation of dorsal root ganglia sensory pathways shared by the bladder and colon^9,20,33^. Further, an altered microbiome has been demonstrated in both IBS and OBS; interestingly, OBS is associated with an altered microbiome in both the bladder and the intestinal tract^9,34,35^. Likewise, an altered epithelial barrier has been demonstrated in both OBS and IBS^9,36^. Lastly, both conditions are associated with depression and anxiety, which may be a shared cause, effect, or interactive process^9,37^. Alternatively, rather than only shared pathophysiology, the association of OBS and IBS could result from crosstalk or cross-sensitization as the bladder and colon share some common sensory pathways^38^. In an animal model, colitis results in increased bladder sensitivity, frequency of urination, and urgency without bladder inflammation^9^.

The main strength of the current study is the standardized symptom reporting in a relatively large group of youth with AP-DGBI. The primary limitation is that this type of study design only allows for assessment of associations and not cause-and-effect relationships. Additionally, the data did not assess the frequency of nighttime urination which may have negatively impacted sensitivity in assessing associations between nighttime urination and gastrointestinal symptoms. Lastly, urinary frequency could have been artificially inflated if patients with loose stools had more frequent stools as patients are likely to void whenever having a stool.

In conclusion, urinary symptoms are common in youth with AP-DGBI. We have demonstrated a specific association of increased urinary frequency and urgency with IBS, specifically in those with predominantly loose stools. If specific bladder and bowel symptoms do share common pathophysiology, bladder symptoms may be an indicator of underlying pathophysiologic targets for treatment of IBS. Further studies are needed to determine to what degree the bladder contributes to symptoms or quality of life, directly or indirectly, and whether the bladder represents a viable treatment target in itself in AP-DGBI patients.

## Methods

This study retrospectively assessed 226 consecutive patients presenting to an abdominal pain clinic at Children’s Mercy Kansas City. All had been diagnosed with an AP-DGBI by a single board-certified pediatric gastroenterologist. Patients ranged in age from 8 to 18 years. All reported pain at least weekly for a minimum of 8 weeks. As part of standard care, all patients completed a symptom questionnaire including gastrointestinal and non-gastrointestinal symptoms. The symptoms included those regarding symptoms utilized in Rome IV criteria. The questionnaire also specifically asked about the presence of increased urinary frequency, nighttime urination, and urinary urgency. The questionnaire was completed within a REDCap database and the database was utilized for analyses. REDCap provides a secure, web-based platform for data acquisition^39^.

The study was approved by the Institutional Review Board (IRB) Children’s Mercy Kansas City which waived Informed consent. All methods were performed in accordance with the relevant guidelines and regulations and in accordance with the Declaration of Helsinki.

### Statistical analysis

SPSS version 23 (SPSS Inc., Chicago, IL) was used to perform statistical analysis. Descriptive statistics are provided. Differences in frequencies were assessed by chi square analysis or the Fischer’s exact test as appropriate for cell sizes. Odds ratios (OR) with 95% confidence intervals (95%CI) were calculated. Differences were considered significant when the 95%CI did not contain 1.0.

## Data Availability

The datasets used and/or analysed during the current study available from the corresponding author on reasonable request.
